# Tanshinone IIA promotes vascular normalization and boosts Sorafenib’s anti-hepatoma activity via modulating the PI3K-AKT pathway

**DOI:** 10.3389/fphar.2023.1189532

**Published:** 2023-06-01

**Authors:** Chengdong Qin, Siyuan Liu, Shiqi Zhou, Xianghou Xia, Jiejie Hu, Yang Yu, Dening Ma

**Affiliations:** ^1^ Department of Breast Surgery, Zhejiang Cancer Hospital, Hangzhou Institute of Medicine (HIM), Chinese Academy of Sciences, Hangzhou, Zhejiang, China; ^2^ Department of Colorectal Surgery, Zhejiang Cancer Hospital, Hangzhou Institute of Medicine (HIM), Chinese Academy of Sciences, Hangzhou, Zhejiang, China; ^3^ Key Laboratory of Prevention, Diagnosis and Therapy of Upper Gastrointestinal Cancer of Zhejiang Province, Hangzhou, China

**Keywords:** tanshinone IIA, hepatocellular carcinoma, angiogenesis, sorafenib, hypoxia

## Abstract

**Introduction:** Angiogenesis is an essential feature of liver cancer. Tumor hypoxia results from abnormal vessel architecture. Numerous studies have sufficiently demonstrated that Tanshinone IIA (Tan IIA) can increase blood flow and enhance microcirculation. The objectives of this study are to: 1 assess the impact of Tan IIA on tumor angiogenesis and architecture, 2 determine the impact of Tan IIA on tumor hypoxia and susceptibility to Sorafenib, and 3 clarify the relevant mechanisms.

**Methods:** CCK8 and flow cytometry measured cell proliferation and apoptosis, respectively. Tube creation assay was used to investigate medication effects on angiogenesis and structure. Drug effects on tumor development, metastasis, and hypoxic tumor microenvironment are assessed in an orthotopic xenograft model of liver tumors. Protein expression was measured by Western blotting and immunohistochemistry.

**Results:** Our results demonstrated that Tan IIA could not reduce tumor proliferation or enhance Sorafenib’s anti-tumor effect *in vitro*. Nevertheless, it can prevent Sorafenib from demolishing the typical vascular structure and aid sorafenib in blocking the recruitment of vascular endothelial cells by liver cancer cells. Although Tan IIA cannot inhibit tumor growth *in vivo*, it can significantly boost Sorafenib’s inhibitory effect on liver cancer, alleviate tumor microenvironment hypoxia, and minimize lung metastasis. This effect may be achieved by reducing HIF-1α and HIF-2α expression via the PI3K-AKT signal pathway.

**Discussion:** Our results reveal the mechanism of Tan IIA in normalizing tumor blood vessels, provide innovative concepts and approaches to overcome chemotherapy resistance, and provide a theoretical basis for the clinical transformation and usage of Tan IIA.

## Introduction

Hepatocellular carcinoma (HCC) is a common type of extremely lethal tumor with a high mortality rate, poor prognosis, difficulties with early diagnosis, and limited treatment options (Kocarnik et al., 2022). Surgical resection is the primary treatment for HCC. However, after surgery, over 70% of patients will redevelop HCC, and the overall 5-year survival rate for HCC patients is just about 30% (Crissien and Frenette, 2014). The FDA-approved medication sorafenib for patients with advanced liver cancer can increase survival time by 3–7 months (Vogel et al., 2021). However, drug efficacy is difficult to sustain for an extended period due to the generation of drug-resistant cells. Therefore, examining the basic mechanisms underlying drug resistance and HCC metastasis and implementing practical intervention strategies would significantly improve the prognosis for HCC patients.

Tissue homeostasis is built on a mature and well-organized vascular network. However, vascular dysfunction is linked to various human diseases, including well-known malignancies (Klein, 2018). The goal of tumor angiogenesis is to create more and more blood vessels to feed growing tumors. This process is ongoing, and abnormal vascular network architecture is present along with it (Lugano et al., 2020). Anomalies in the tumor vascular system’s structure have a significant impact on tumor pathogenesis, growth, metastasis, and responsiveness to anti-cancer therapy. Abnormalities in the tumor’s vasculature can lead to increased vascular permeability, inadequate regional perfusion, and enhanced interstitial fluid pressure (IFP), which can result in localized hypoxia and acidosis inside the tumor (Teleanu et al., 2019).

On the one hand, hypoxia can promote the production of several growth factors and activate oncogenes that accelerate invasive tumor development and metastasis (Jiang et al., 2020). On the other hand, hypoxia and an acidic microenvironment impair the cytotoxicity of tumor-infiltrating immune cells, thereby promoting tumor aggressiveness (Albini et al., 2018). Moreover, unbalanced tumor blood supply and elevated IFP allow anti-tumor drugs to be over-concentrated in areas with adequate blood supply and unable to reach areas with inadequate blood supply. At the same time, hypoxia is a well-known mediator of cancer cell resistance to cytotoxic drugs, thereby diminishing the therapeutic effect of anti-tumor medications (Lugano et al., 2020). Thus, structurally aberrant tumor blood vessels are intimately associated with tumor growth, metastasis, and treatment resistance.

Traditional Chinese medicine regularly uses danshen, also known as *Salvia miltiorrhiza* (the dry root of *S. miltiorrhiza*, Labiatae), to treat cardiovascular and vascular diseases (Huang et al., 2022). In various human cancer cells, Tan IIA, a fat-soluble component of *S. miltiorrhiza,* inhibits proliferation, promotes apoptosis, and reverses multidrug resistance (Lin et al., 2018; Zhou et al., 2020; Li et al., 2021; Li et al., 2021). Prior research has demonstrated that Tan IIA can also affect tumor angiogenesis, particularly in controlling hypoxia (Zhang et al., 2022). Combination therapies, which combine many treatments, have recently emerged as a potentially helpful approach to cancer control. In the present study, the underlying mechanisms of Tan IIA’s effects on tumor vascular integrity and permeability as well as Tan IIA’s combined effects with Sorafenib on the progression and metastasis of liver cancer were examined.

## Materials and methods

### Cell lines culture

Because different cell lines can have varying sensitivities to drugs and different gene expression profiles, we utilized two types of HCC lines in the functional assays to increase the robustness and reliability of our findings. Human HCC cell line Huh7 cells were obtained from the Cell Bank of the Chinese Academy of Sciences (Shanghai, China), and human HCC cell line MHCC97H cells were obtained from the Liver Cancer Institute of Fudan University. Primary human umbilical vein endothelial cells (HUVECs) were purchased from the American Type Culture Collection (ATCC). All HCC cell lines were grown in Dulbecco’s modified Eagle’s medium (DMEM; BasalMedia, Shanghai, China) supplemented with 10% fetal bovine serum (FBS), 100 U/mL penicillin, and 50 mg/mL streptomycin. Primary umbilical vein endothelial cells were grown in Endothelial Cell Medium (ECM) with 5% FBS, 1% ECGF, and 1% penicillin/streptomycin (P&S, Gibco, United States).

### Regents and antibodies

*In vitro* study, Tan IIA monomer (purchased from the MedChemExpress), a lyophilized powder with a purity of 99.99%, firstly dissolved in dimethyl sulfoxide (DMSO) and then diluted with PBS to the required concentration, was used in the assays. Tan IIA (sulfotanshinone sodium injection, 5 mg/mL), available commercially from the first Biochemical Pharmaceutical Co. Ltd. in Shanghai, China, was utilized in an *in vivo* experiment. Sorafenib was purchased from Selleckchem Inc. (Houston, TX, http://www.selleckchem.com). Antibodies used for immunofluorescence and immunoblotting were as follows: rabbit anti-human monoclonal HIF-1α (CST), rabbit anti-human monoclonal HIF-2α (CST), rabbit anti-human monoclonal Phospho-PI3 Kinase p85/p55 (Tyr467/Tyr199) (ZENBIO, China), rabbit anti-human monoclonal PI3 Kinase p85 alpha (ZENBIO, China), rabbit anti-human monoclonal Phospho-AKT (Ser473) (ZENBIO, China), rabbit anti-human monoclonal AKT (ZENBIO, China), rabbit anti-human monoclonal Phospho-mTOR (Ser2448) (ZENBIO, China), rabbit anti-human monoclonal mTOR (ZENBIO, China).

### Experimental groupings

The drug intervention subgroups for cell proliferation assay, flow cytometry assay, and cell migration assay were: Tan IIA 10 μM, Sorafenib 5 μM, and Tan IIA 10 μM plus Sorafenib 5 μM. And DMSO (0.02%) was used as a negative control. The drug intervention subgroups for tube formation assay were: Tan IIA 5 μM, Tan IIA 10 μM, Sorafenib 5 μM, Tan IIA 5 μM plus Sorafenib 5 μM, and Tan IIA 10 μM plus Sorafenib 5 μM. DMSO (0.02%) was used as a negative control. For the *in vivo* assay, mice were divided into four groups (each *n* = 6) and received daily injections of normal saline (NS), Sorafenib (30 mg/kg/d), Tan IIA (10 mg/kg/d), and Sorafenib (30 mg/kg/d) plus Tan IIA (10 mg/kg/d).

### Cell proliferation assay

Prior to the assay, hepatocellular carcinoma cells (including Huh7 and MHCC97H cells) and HUVECs were treated with Tan IIA 10 μM for 72 h. Plant hepatocellular carcinoma cells and HUVECs at a density of 5,000 cells per well in 100 μL of complete growth medium (DMEM or ECM) in 96-well plates. After cell attachment, 100 μL of the diluted drug-containing medium is added to each well. Incubate the cells for the desired amount of time (0, 24, 48, and 72 h) at 37°C and 5% CO_2_ in a humidified atmosphere. The Cell Counting Kit-8 (CCK-8; NCM Biotechnology, China) was used for cell proliferation assays. Briefly, 10 μL of CCK-8 reagent was added to each well, followed by 2 h of 37°C incubation. The optical density was determined at a wavelength of 450 nm.

### Flow cytometry assay

In a six-well plate, 1.5×10^6^ HUVECs were seeded and allowed to adhere. Then, 3 mL of the diluted drug-containing medium is added to each well. After treatment, cells were collected and washed with cold PBS buffer. The cells were resuspended in a binding buffer and stained for 30 min at 4°C and in the dark with 5 μL Annexin V-FITC and 5 μL pro-podium iodide (Beyotime, Shanghai, China). To eliminate excess dyes, the labeled cells were washed three times with binding buffer and then resuspended in 500 μL binding buffer. Within 1 hour, the proportion of apoptosis was analyzed by flow cytometry (BD, FACSCalibur, United States).

### Tube formation assay

Endothelial cells (2×10^5^ cells/well) were seeded into a matrix-coated (50 μL/hole) 48-well plate. In each well, diluted drug-containing medium was added. After 6 h, we examined the tube shape with an inverted microscope (Olympia America, Inc., Center Valley, PA) and calculated the grid area to determine the degree of tube formation.

### Cell migration assay

Transwell analysis was used to evaluate quantitative cell migration (Boyden Chambers; Corning Inc.). Initially, HCC cells were pretreated for 72 h with the diluted drug-containing medium. Subsequently, an equal number of HCC cells from each subgroup were added to the bottom chamber of the TRANSWELL plate and allowed to adhere for about 4 h. Then, 5×10^4^ HUVECs were introduced to the upper chamber of each well of 24-well plates having membranes with an 8.0 μm pore size, and the lower chamber was filled with DMEM containing 5% FBS. The cells migrated over 24 h at 37°C. Crystal violet is applied to stained cells that have reached the membrane’s bottom. Finally, at ×100 magnification, calculate the number of stained cells in 3 random fields.

### Xenograft model of human HCC in nude mice

Male BALB/c nude mice were acquired from Shanghai SLAC Laboratory Animal Co. Ltd. (Shanghai, China) and housed in pathogen-free environments. For the xenotransplantation model, 5×10^6^ Huh7 and MHCC97H cells were injected subcutaneously into the left upper abdomen of three nude mice (4 weeks old). One month later, the subcutaneous tumor tissue was removed and sliced into 1× mm^3^ pieces; then, a tumor piece was implanted within the liver’s left lobe according to standard procedures described previously (six per group) (Sun et al., 1996). One week later, Sorafenib (30 mg/kg/d) and Tan IIA (10 mg/kg/d) were injected intragastrically and intraperitoneally into the null mice. Mice were killed after 40 days, and tumor tissues and lungs were collected, photographed, and weighed. The tumor volume is computed using the following formula: tumor volume (mm^3^) = (maximum diameter× minimum diameter^2^)/2. All operations completely adhere to the Laboratory Animal Care Committee’s set criteria.

### *In vivo* hypoxia probe test

Pimonidazole hydrochloride (60 mg/kg) was injected into the tail veins of the nude mice 90 min before they were euthanized. After the mice were euthanized, their liver tumors were excised, fixed in paraformaldehyde, and embedded in paraffin. Each group’s hypoxic area was detected using immunohistochemistry with the help of the hypoxia probe kit (Hypoxyprobe™-1 kit, Hypoxyprobe Inc., Burlington, MA), and their respective IOD values were evaluated using image pro plus software.

### Western blotting

Using ice-cold RIPA buffer (150 mM NaCl, 50 mM Tris-HCl, pH 8.0, 0.1% SDS, 1% Triton X-100, 0.1% SDS, 1% Triton X-100) containing protease and phosphatase inhibitors, cells were lysed. Each group’s proteins were separated by SDS-PAGE and transferred to polyvinylidene fluoride (PVDF) membranes in equal amounts. The membranes were blocked with 5% fat-free milk for 1 hour. Then, membranes were cut horizontally by using the straightedge and treated overnight at 4°C with the corresponding primary antibody. The membranes were then rinsed three times in Tris-buffered saline with Tween-20 (TBST) and incubated for 1 hour at room temperature with the matching HRP-conjugated secondary antibody. Blots were examined with enhanced chemiluminescence (ECL) detection kit (NCM Biotech, China) and visualized using a chemiluminescence system (Bio-Rad ChemiDoc™ Imaging System), analyzed with the ImageJ software (https://imagej.nih.gov).

### Statistical analysis

Data were examined using GraphPad Prism (version 9.5.0, GraphPad Software, San Diego, CA, United States). The Student’s t-test was used to assess differences between the two groups, while one-way or two-way ANOVA was used to compare more than two groups. For the *in vivo* testing, the unpaired *t*-test was employed to assess differences in tumor volume.

## Results

### Tan IIA can diminish Sorafenib’s lethal activity on normal vascular endothelial cells

**FIGURE 1:**
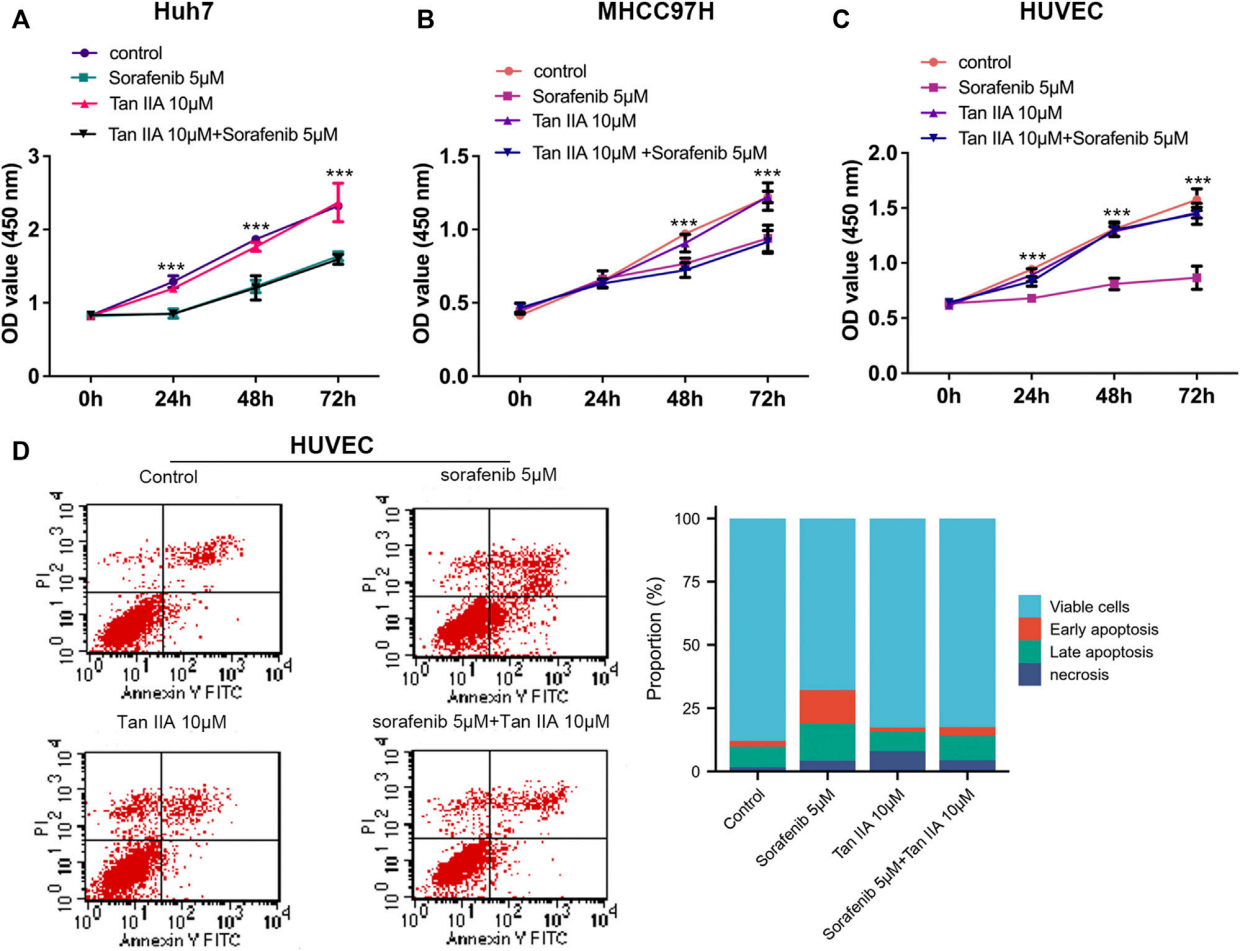
Effects of Tan IIA and Sorafenib on the proliferation of HUVECs and HCC cells. **(A, B)** CCK8 was utilized to determine the effect of Tan IIA 10 μM in combination with Sorafenib 5 μM on the proliferation of liver cancer cells **(C)** Effect of Tan IIA 10 μM and Sorafenib 5 μM on the Proliferation of HUVECs. The results are reported as the mean standard deviation (S.D.). The significant difference was tested by two-way ANOVA. ***p < 0.001 **(D)** Flow cytometry was utilized to determine the effect of Tan IIA in combination with or without Sorafenib on the apoptosis of HUVECs. The bar chart shows the percentage of viable cells, early apoptosis, late apoptosis, and necrosis in each subgroup.

CCK-8 tests were used to determine the viability of liver cancer cells (Huh7 and MHCC97H) and HUVECs after being treated with Tan IIA 10 μM in combination with or without Sorafenib 5 μM, aiming to investigate their influence on the proliferation of liver cancer cells and HUVECs. CCK8 experiments were measured at 0, 24, 48, and 72 h after drug treatment. As shown in Figures 1A, B, Sorafenib dramatically inhibits the proliferation of liver cancer cells, but Tan IIA does not influence the growth of liver cancer cells, regardless of whether it is combined with Sorafenib; and these results were consistent with previous research (Wang et al., 2012). Although sorafenib could also substantially inhibit HUVECs proliferation, Tan IIA can lessen this effect (Figure 1C). Flow cytometry further demonstrated that the number of early and late apoptotic HUVECs was significantly higher in the sorafenib group than in the Tan IIA group and Tan IIA plus Sorafenib groups (Figure 1D). These findings revealed that Tan IIA did not augment Sorafenib’s inhibitory effect on liver cancer cell proliferation *in vitro*. Nevertheless, it diminished Sorafenib’s cytotoxic effect on normal vascular endothelial cells.

### Tan IIA mitigates the sorafenib-induced deterioration of normal vascular integrity

**FIGURE 2:**
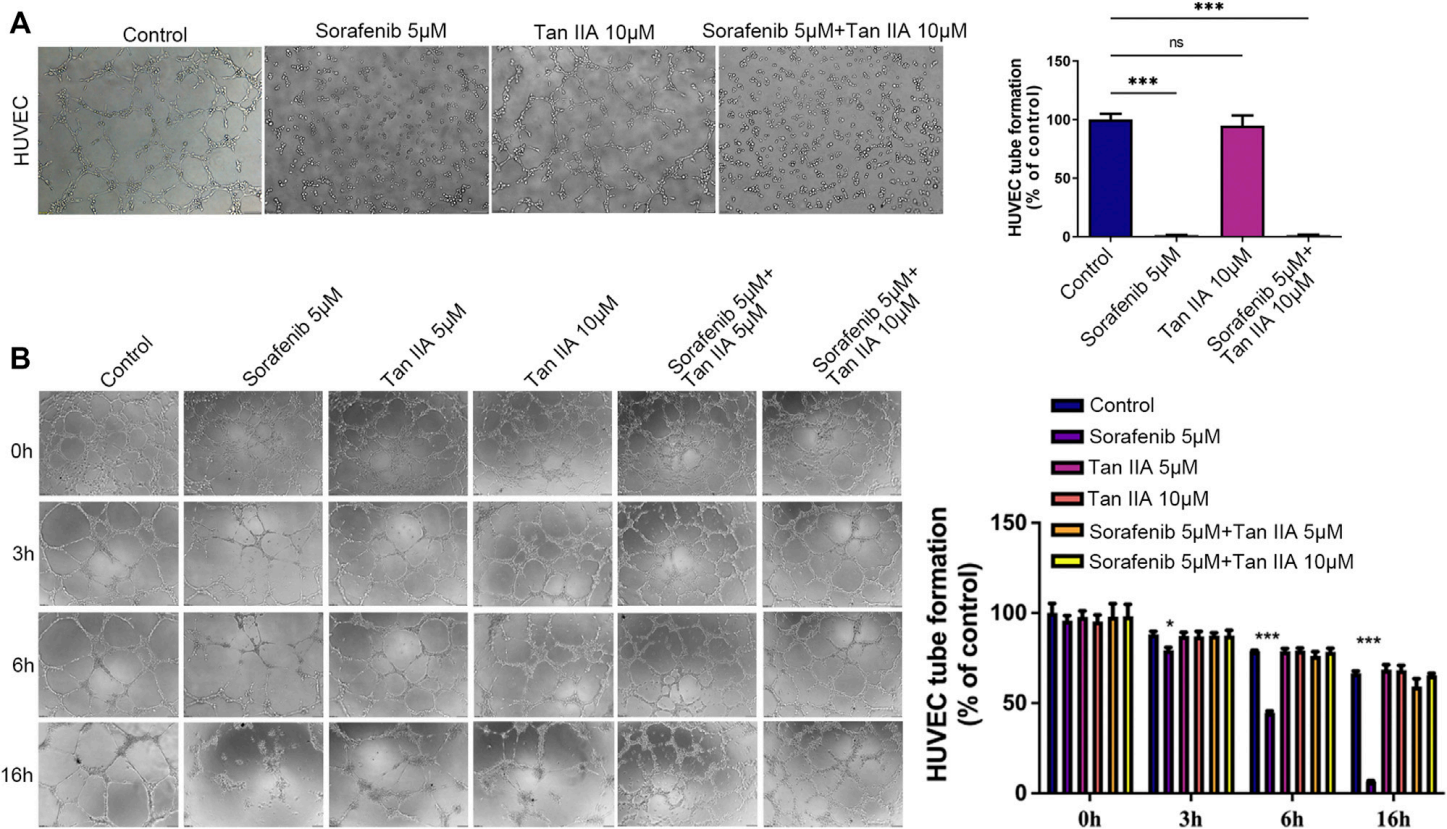
Effect of Tan IIA in Combination with Sorafenib on Angiogenesis and Vascular Integrity *in vitro* by Tubular Formation Test. **(A)** The influence of tan IIA 10 μM with or without Sorafenib 5 μM on angiogenesis. Magnification: ×100. In three different fields of view, the number of tubes was counted. The significant difference was tested by one-way ANOVA. ns: no significance. ***p < 0.001 **(B)** The effect of Tan IIA coupled with or without Sorafenib on vascular integrity at 0h, 3h, 6, and 16 h. Magnification, ×100. In three different fields of view, the number of tubes was counted. The significant difference was tested by one-way ANOVA. *p < 0.05. ***p < 0.001.

Through an experiment involving tubule development, we investigated how the presence of Tan IIA affected Sorafenib’s inhibitory effect on angiogenesis. According to the results, neither the group receiving Sorafenib nor the group receiving Sorafenib in combination with Tan IIA produced formal circular formations (Figure 2A). Then, we evaluated the effect of Sorafenib and Tan IIA on the formed blood vessels *in vitro*. After forming a mature vascular ring structure 3 hours later, replace the supernatant with a medium containing either Sorafenib 5 μM, Tan IIA 5 μM and 10 μM, or Sorafenib 5 μM combined with Tan IIA 5 μM and 10 μM. After 3 hours of observation, the difference between the groups was not particularly evident, but after 6 hours, the destruction of the circular structure of blood vessels in the sorafenib group was much greater than in the other groups (Figure 2B). The maximum time to preserve the annular configuration of blood arteries was about 16 h; in the sorafenib-treated group, the annular configuration of blood vessels could not be seen; however, in the combination-treated group, partial annular structures were still evident (Figure 2B). These data suggest that Tan IIA does not affect Sorafenib’s ability to suppress angiogenesis *in vitro* but prevents Sorafenib from destroying normal vascular integrity.

### The combination of Tan IIA and sorafenib further inhibits the recruitment of vascular endothelial cells by tumor cells

**FIGURE 3:**
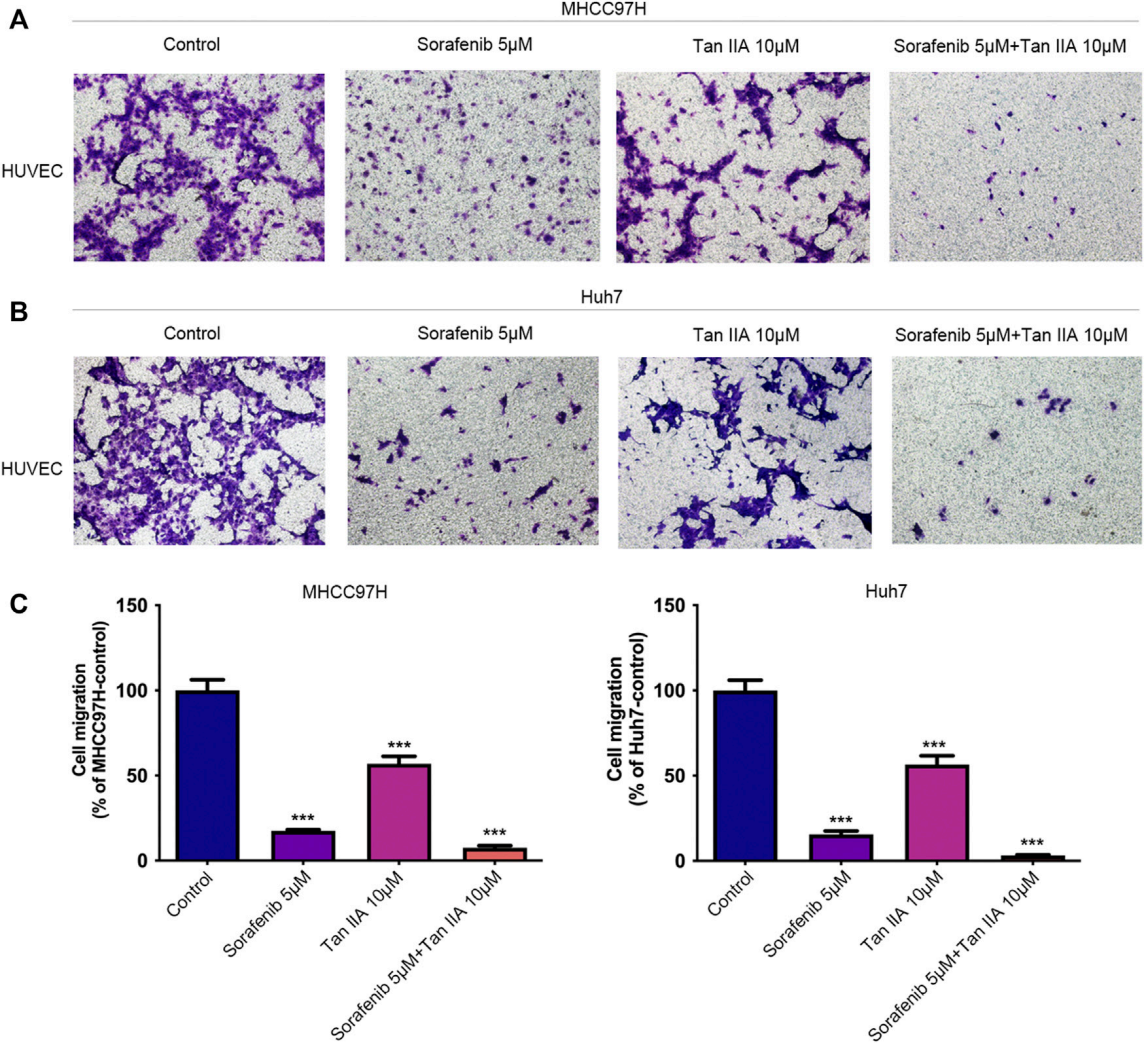
Effect of the combination of Tan IIA and Sorafenib on the capacity of liver cancer cells to recruit HUVECs. **(A)** The effect of tan IIA 10 μM in combination with or without Sorafenib 5 μM on MHCC97H’s capacity to recruit HUVECs was determined by the transwell assay **(B)** The effect of tan IIA 10 μM in combination with or without Sorafenib 5 μM on Huh7’s ability to recruit HUVECs was assessed using the transwell assay. **(C)** The number of cells that passed through the membrane was counted and compared in the graphs; the data are expressed as the mean standard deviation (*n* = 3); one-way ANOVA was used to evaluate significance. ***p < 0.001.

The recruitment of vascular endothelial cells by tumor cells is one of the fundamental processes of tumor angiogenesis (Kretschmer et al., 2021). We examined the effects of different drug groups using the transwell assay to determine how well tumor cells could attract vascular endothelial cells. After treatment with Tan IIA or Sorafenib, especially in the group that received both drugs, liver cancer cells were much less able to recruit other cells, especially vascular endothelial cells, into their colony (Figures 3A, B). This reduction was measured compared to the control group (Figure 3C). Based on these findings, Tan IIA may enhance sorafenib’s effect in inhibiting tumor blood vessel formation when combined. However, the underlying mechanism is unknown and requires further study.

### Tan IIA can potentiate the inhibition activity of sorafenib on liver cancer growth and metastasis *in vivo*

**FIGURE 4:**
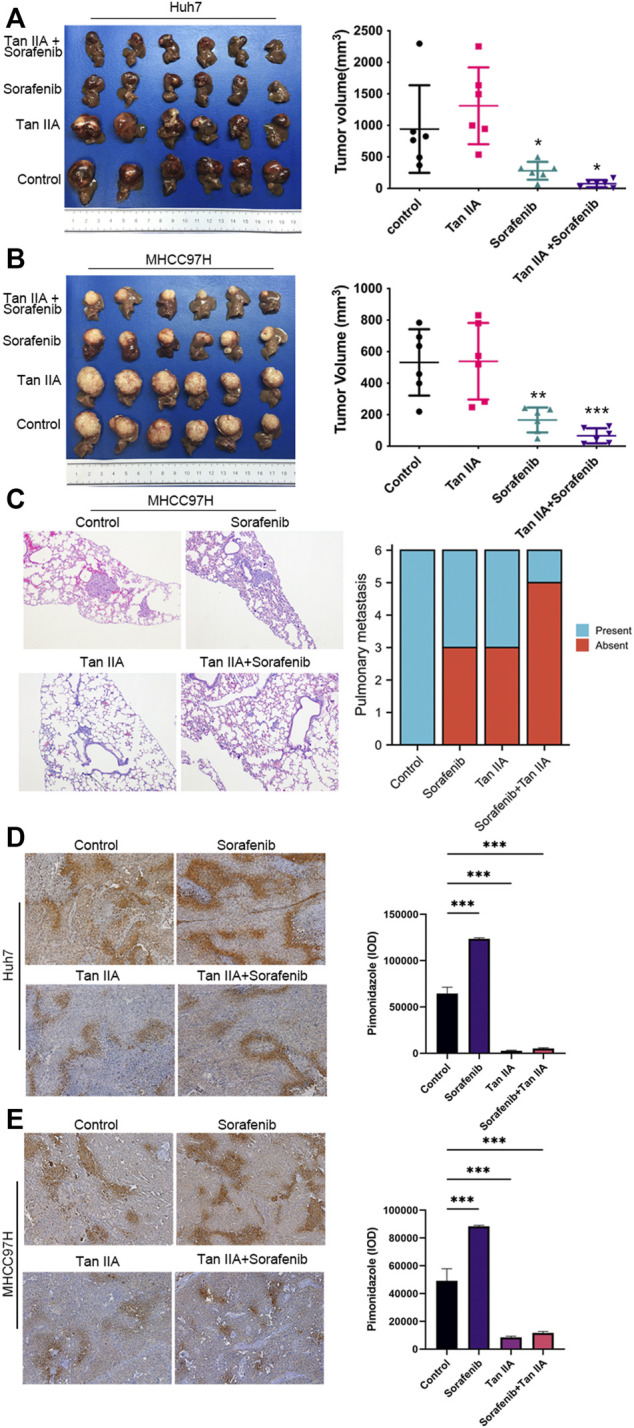
The effect of tan IIA and Sorafenib on the growth, lung metastasis, and hypoxia of liver cancer *in vivo*. **(A, B)** Huh7 and MHCC97H cells were employed to create a xenograft orthotopic tumor model. For intervention, tan IIA and/or Sorafenib were utilized. Each group’s tumor volume is computed using the formula: tumor volume (mm^3^) = (maximum diameter× minimum diameter^2^)/2, and compared in the chart. Examine the significance of the difference using the unpaired *t*-test. *p < 0.05, **p < 0.01, ***p < 0.001. **(C)** Images of representative HE staining for lung metastases. Magnification, ×100. The number of mice with and without lung metastatic nodules was counted in the graph (6 mice in each group). **(D, E)** Representative pictures of pimodazole region in control, tan IIA, sorafenib, tan IIA + sorafenib groups. Magnification: ×100. The pimonidazole region was quantitated based on integrated optical density (IOD). Results were expressed as mean ± S.D. The significant difference was tested by one-way ANOVA. ***p < 0.001.

As there are additional factors such as blood vessels and extracellular matrix *in vivo*, the Xenograft model provides more accurate modeling of the complex tumor microenvironment to evaluate the impact of Tan IIA on liver cancer. Therefore, we examined the effects of Tan IIA, Sorafenib, and their combination on the growth of liver cancer *in vivo* using the nude mouse hepatoma *in situ* tumor model. There was no significant difference in tumor volume between the Tan IIA group and the control group; however, the combination of Sorafenib and Tan IIA resulted in the smallest tumor volume (Figures 4A, B). Besides, all animals in the MHCC97H control group exhibited lung metastases, compared to three mice in the sorafenib group, three mice in the Tan IIA group, and one in the mouse in the Sorafenib plus Tan IIA group (Figure 4C). These results indicate that Tan IIA alone does not affect tumor growth *in vivo*. However, the combination of Tan IIA and Sorafenib can further suppress tumor proliferation and lung metastasis.

### Tan IIA can alleviate the hypoxemia in liver cancer caused by sorafenib

Using a hypoxia probe, we simultaneously determined the degree of hypoxia in each tumor group. The results demonstrated that intra-tumor hypoxia was more severe in the sorafenib alone group than in other groups. In contrast, the combination of Tan IIA and Sorafenib dramatically reduced intra-tumor hypoxia in the group (Figures 4D, E). These data indicate that Tan IIA has a considerable ameliorating impact on sorafenib-induced hypoxia in tumors.

### Tan IIA inhibits HIF-1α and HIF-2α expression *in vivo* concurrently

**FIGURE 5:**
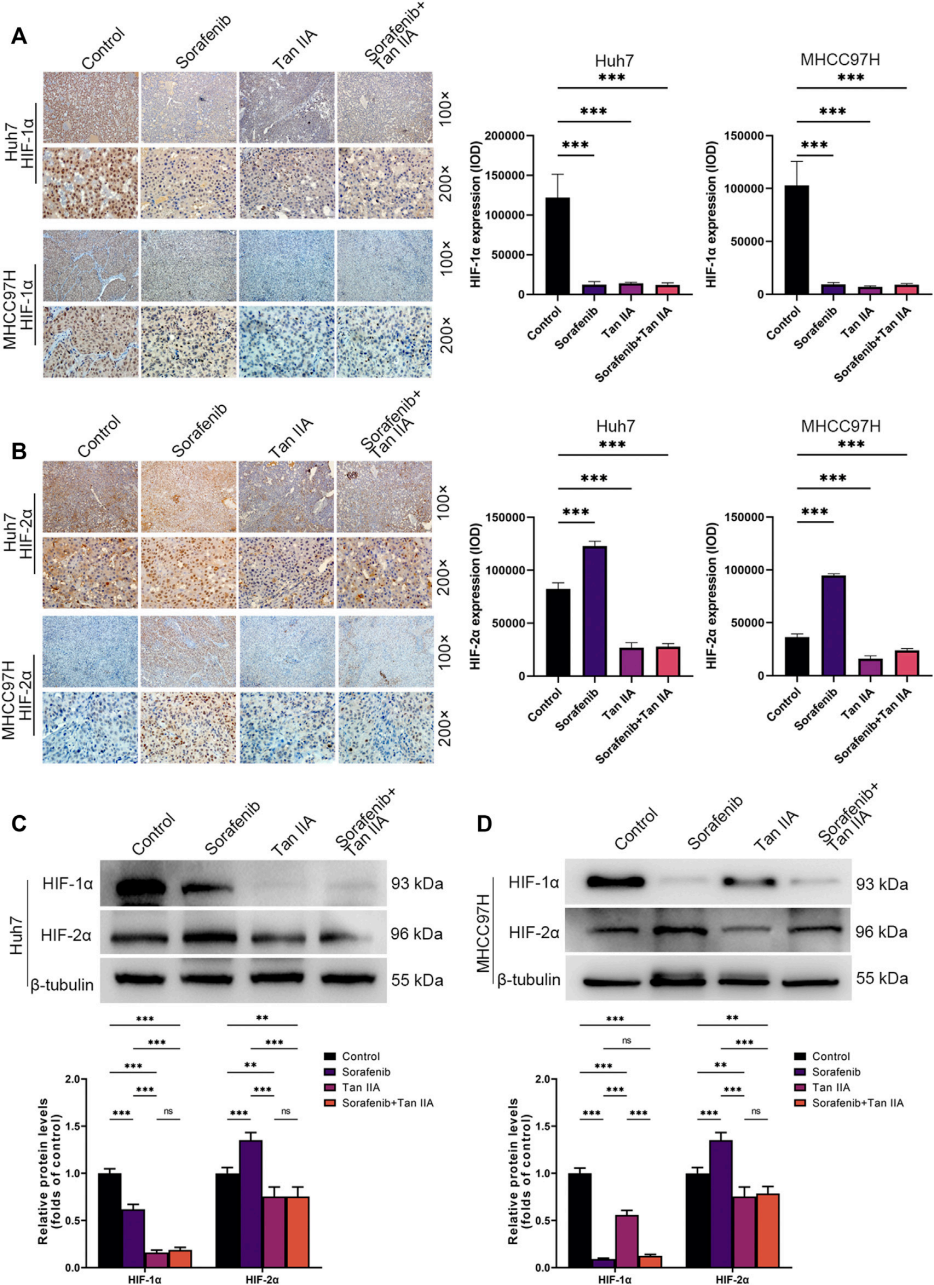
*In vivo* influence of tan IIA and Sorafenib on HIF-1α and HIF-2α expression in HCC. **(A)** Representative immunohistochemistry pictures of HIF-1α in each intervention group of the Huh7 and MHCC97H orthotopic xenograft tumor models are displayed. Magnification: ×100 (upper), ×200 (lower). HIF-1α expression was quantitated based on integrated optical density (IOD). Results were expressed as mean ± S.D. The significant difference was tested by one-way ANOVA. ***p < 0.001. **(B)** On show are immunohistochemical images of HIF-2α in the Huh7 and MHCC97H orthotopic xenograft tumor models from each intervention group. Magnification: ×100 (upper), ×200 (lower). HIF-2α expression was quantitated based on integrated optical density (IOD). Results were expressed as mean ± S.D. The significant difference was tested by one-way ANOVA. ***p < 0.001 **(C, D)** Analysis of HIF1-α and HIF2-α expression in HCC tissues derived from each intervention group of the Huh7 and MHCC97H orthotopic xenograft tumor models using Western blotting. All data for the densitometric analysis of the western blots are presented as mean ± S.D. (*n* = 3). The density of each band was normalized to β-tubulin. Significance was determined using two-way ANOVA. **p < 0.01, ***p < 0.001.

We also determined the hypoxia indicators HIF-1α and HIF-2α expression in each tumor subgroup by immunohistochemistry. The results demonstrated that Sorafenib could restrict the expression of HIF-1α while simultaneously stimulating the expression of HIF-2α *in vivo* (Figures 5A, B). Tan IIA, however, can simultaneously decrease HIF-1α and HIF-2α expression *in vivo* (Figures 5A, B). Moreover, Tan IIA and sorafenib combination did not raise HIF-2α expression in tumors (Figure 5B). Meanwhile, Western blot results show that Sorafenib could decrease the expression of HIF-1α while boosting the expression of HIF-2α, and that the addition of Tan IIA could prevent this from occurring (Figure 5C, D). The aforementioned findings show that Tan IIA can block the sorafenib-induced conversion of the tumor-dependent HIF-1α pathway to the HIF-2α pathway.

### Tan IIA modulates the activity of the PI3K-AKT signaling pathway

**FIGURE 6:**
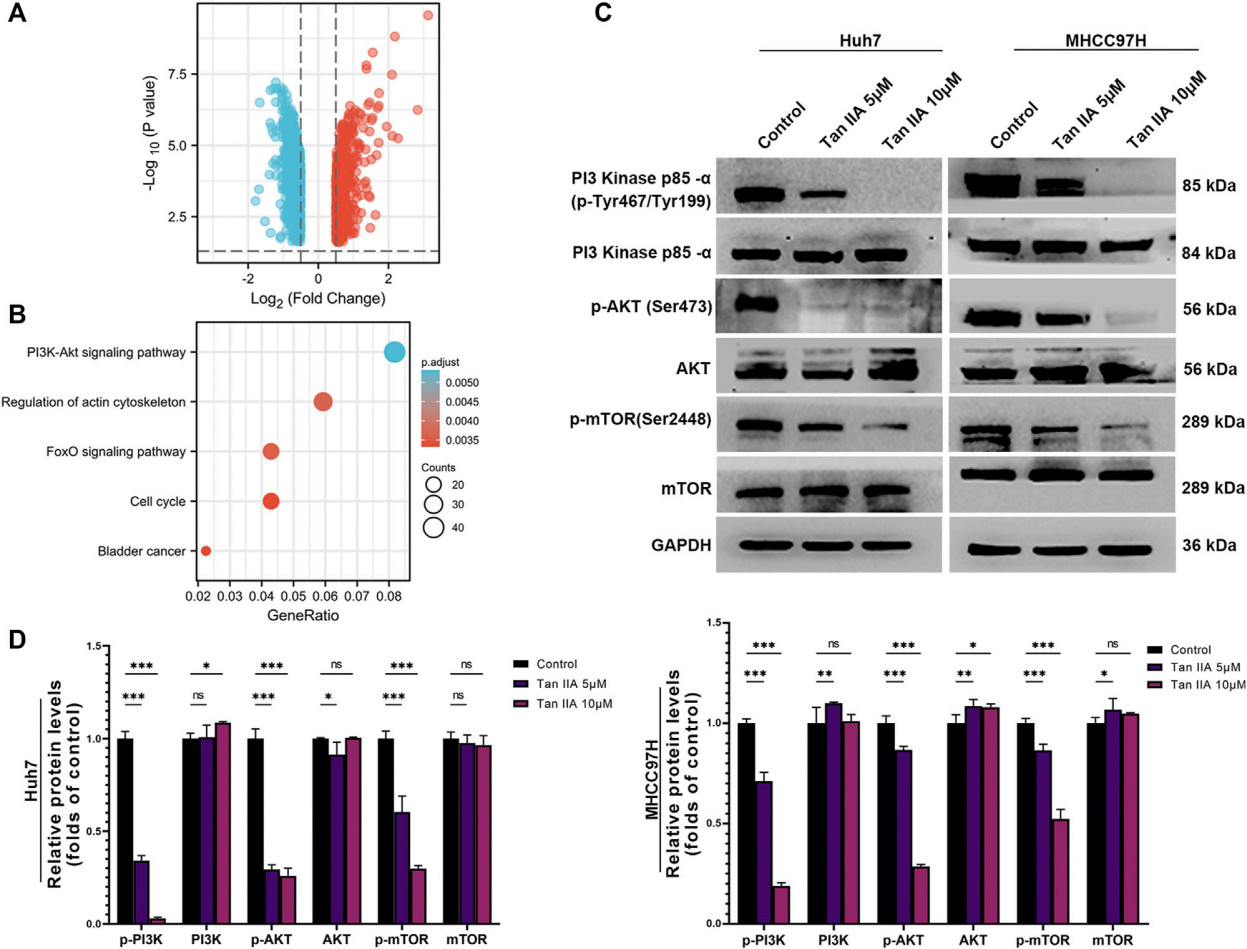
Effects of tanshinone on gene expression and signal transduction in cancer cells. **(A)** The volcanic map depicts the up (red) or down (blue) genes in tumor cells before and after therapy with tan IIA (GSE85871), as determined by DEGseq analysis. **(B)** The outcomes of a KEGG pathway study for tan IIA. The color denotes significance [- log10 (*p*-value)], while the size of the circle represents the abundance of genes in the relevant annotation. **(C)** The phosphorylation levels of PI3K, AKT, and mTOR in Huh7 and MHCC97H cells after 24 h of treatment with 5 μM and 10 μM tan IIA were analyzed by Western blotting **(D)** All data for the densitometric analysis of the western blots are presented as mean ± S.D. (*n* = 3). The density of each band was normalized to GAPDH. Significance was determined using two-way ANOVA. ns: no significance. *p < 0.05, **p < 0.01, ***p < 0.001.

We used the DESeq2 method (Love et al., 2014) and the GEO database (GSE85871) to investigate gene expression changes in tumor cells before and after treatment with Tan IIA. Our goal was to determine which signaling pathways Tan IIA could potentially modulate. Following treatment with Tan IIA, the findings indicated that 602 genes had decreased expression levels, while 508 genes had increased expression levels (Log2FC −0.5 or >0.5; *p*-value 0.05; Figure 6A). Subsequent KEGG enrichment analysis indicates that most of these differential genes are enriched in the PI3K-AKT signaling pathway (Figure 6B). The results of the Western blot analysis also revealed that phosphorylation levels of PI3K, AKT, and mTOR decreased dramatically with increased Tan IIA concentration (Figures 6C, D).

## Discussion

The rapid development and metabolism of tumor cells are supported by angiogenesis, which makes HCC highly vascularized and vulnerable to significant bleeding during surgery (Viallard and Larrivée, 2017). Several macro and micro phenotypic differences exist between cancer-associated blood vessels and their normal counterparts. Anatomically, the tumor vasculature often has a convoluted and dilated appearance. Its structural faults prohibit blood arteries from adequately supplying oxygen and provide a hypoxic milieu, including disordered branching and crosslinking as well as an abnormally enlarged lumen (Abou Khouzam et al., 2020; Bhat et al., 2021). The vascular endothelial cells in the tumor had an irregular and disordered appearance at the cellular level. Tumor cells can manufacture hydrolases to promote VE-cadherin disintegration, in contrast to normal vascular endothelial cells that require VE-cadherin attachment. This triggers weak adhesion between tumor vascular endothelial cells and disruption of barrier integrity (Zanotelli and Reinhart-King, 2018). Additionally, blood vessel permeability may also be increased by the production of several inflammatory chemicals by tumor cells (Wang et al., 2021). Moreover, a further increase in vascular permeability is the instability of the vascular wall caused by the vascular pericytes’ inadequate ability to control blood flow due to the loose adhesion of vascular endothelial cells (Matuszewska et al., 2021). Because of these factors, there is a higher chance that tumor cells will penetrate the circulatory system, spread to distant tissues, and bleed or become hypoxic.

Physical compression, an imbalanced signal between pro- and anti-angiogenic molecules, can result in aberrant or chaotic tumor vasculature, poor blood perfusion, and reduced oxygen uptake (Huang et al., 2021). Blood flow is impacted by several variables, including tumor growth stage, location, area, and primary tumor, and the situation deteriorates as the cancer progresses (Huang et al., 2021). Reduced blood flow can create a microenvironment with high acidity and low oxygen levels. An atypical tumor microenvironment can help tumor cells evade the immune system, increase their invasion and metastasis capacity, and motivate them to adapt to this abnormal milieu. The ability of immune cells to eradicate tumor cells, the activity of T lymphocytes, and the presentation of tumor antigens by dendritic cells by hypoxia and an acidic microenvironment can all be compromised (Huang et al., 2021). Additionally, hypoxia can increase VEGF and TGF synthesis, which inhibits immune cell function (Guo et al., 2020). Moreover, hypoxia can activate HIF, which in turn stimulates the synthesis of the immunological checkpoint protein PD-L1, allowing tumor cells to elude the immune system (El Hafny-Rahbi et al., 2021). In addition, hypoxia can promote the production of tumor stem cells, rendering tumors more resistant to radiation and other types of therapy (Emami Nejad et al., 2021). These results indicate a strong correlation between hypoxia and poor prognosis of malignancies. Therefore, to achieve normalization of the microenvironment, it is a possible therapy strategy to reconstruct the natural shape of tumor blood vessels and improve the hypoxic situation.

Sorafenib is an oral multikinase inhibitor that inhibits the tyrosine kinase activity of VGFR-2, VEGF-3, PDGFR, KIT, FLT-3, RET, and other receptors, as well as the serine/threonine kinase activity of RAF-1 and B-RAF (Keating, 2017). Thus, Sorafenib could directly decrease the proliferation of liver cancer cells by blocking the MAPK signaling pathway. By affecting VEGFR, it can also restrict the development of blood vessels in liver cancer, thereby decreasing the progression of liver cancer (Zhu et al., 2017). Although Sorafenib has produced promising results in treating liver cancer, only about 30% of patients can benefit from sorafenib treatment, and many of them develop drug resistance within 6 months (Tang et al., 2020). Epithelial-mesenchymal transition (EMT) of liver cancer cells and subsequent treatment resistance may result from sorafenib inhibition on liver cancer blood vessels, which may worsen microenvironmental hypoxia (Xia et al., 2020). Hypoxia caused by Sorafenib, on the other hand, can boost the accumulation of HIF-1α and HIF-2α in the nucleus, hence accelerating oncogene transcription and enhancing the tolerance of hepatoma cells to Sorafenib (Méndez-Blanco et al., 2018). In addition, hypoxia induced by Sorafenib can hasten the transcription of stem cell regulatory genes and the expression of stem cell markers, increasing the stemness properties of liver cancer cells and promoting their invasion and metastasis as well as their treatment resistance (Xia et al., 2020). By increasing the coverage rate of perivascular cells and restoring the connection structure between endothelial cells, it is possible to promote the maturation and normalization of tumor blood vessels, which can enhance tumor vascular perfusion, increase the oxygen content in the tumor, and prevent the spread of tumor cells and the creation of drug-resistant cells owing to hypoxia. Additionally, improved delivery of chemotherapy drugs to the tumor site via restored tumor blood vessels may increase the cytotoxic effects of the drugs on tumor cells.

Tan IIA is a diterpene quinone that exhibits anti-inflammatory, anti-atherosclerotic, anti-myocardial hypertrophy and prevention of angina pectoris and palpitation infarction properties (Ansari et al., 2021). Previous studies have demonstrated that Tan IIA suppresses cancer proliferation, induces apoptosis, regulates the cell cycle, modifies signal pathways, and overcomes therapeutic resistance (Liu et al., 2019; Fang et al., 2020). Chien et al. found that Tan IIA inhibits liver cancer cell proliferation by altering the expression of Caspase3 and CD31 (Chien et al., 2012). Tan IIA combined with trans-resveratrol has been shown by Chang et al. to enhance apoptosis of liver cancer cells and arrest the cell cycle in the G1 phase (Chang et al., 2014). By up-regulating the expression of VEGFR and PDGFR, Wang et al. found that Tan IIA could enhance blood vessel integrity, thereby reducing tumor cell metastasis through partial liver cancer resection (Wang et al., 2012). To summarize, Tan IIA has a variety of mechanisms in which it can exert its anti-tumor effect.

Our study found that Tan IIA did not enhance Sorafenib’s inhibitory effect on hepatocellular carcinoma cells *in vitro*, but it did reduce Sorafenib’s lethal effect on healthy normal vascular endothelial cells. In addition, Tan IIA also prevents Sorafenib from altering healthy normal vascular integrity while preserving the drug’s ability to inhibit angiogenesis. We also noticed that Tan IIA alone had no effect on tumor growth *in vivo* experiments, but Tan IIA plus Sorafenib significantly inhibited tumor growth and lung metastasis. Furthermore, to determine how Tan IIA affected hypoxia in hepatocellular cancer, we used hypoxia probes in our investigation. It has been shown that hypoxia caused by Sorafenib can activate hypoxia-inducible factors, which may help explain why hepatocellular carcinoma cells are resistant to Sorafenib. In addition, A switch from an HIF1α-dependent pathway to an HIF2α–dependent system is also brought about by sorafenib, thus resulting in a more robust and adequate tolerance of hepatocellular cancer cells to Sorafenib (Zhu et al., 2017; Méndez-Blanco et al., 2018). We next used immunohistochemistry to measure the expression of HIF-1α and HIF-2α, and the results showed that Tan IIA had inhibitory effects on both HIF-1α and HIF-2α *in vivo*. Therefore, we believe that the following mechanisms explain why the combination of Tan IIA and sorafenib can boost anti-tumor cell proliferation and metastasis of sorafenib *in vivo* but not *in vitro*: Tan IIA could promote maturation and normalization of tumor blood vessels, which can enhance tumor vascular perfusion, increase oxygen in the tumor microenvironment, and reduce the development of sorafenib-resistant cells and prevent the spread of tumor cells under hypoxia. In addition, restoring the normal vasculature structure by Tan IIA may enhance sorafenib delivery to the tumor site, enhancing sorafenib’s cytotoxic effect on tumor cells. Furthermore, by reducing the expression of HIF-1α and HIF-2α, Tan IIA may also increase the susceptibility of hepatocellular carcinoma cells to the cytotoxic effects of Sorafenib. In short, Tan IIA boosts sorafenib’s antitumor effect by remodeling and restoring the normal structure of tumor blood vessels, rather than by direct cytotoxic action. Besides, we also analyzed the changes in gene expression in tumor cells before and after Tan IIA treatment using the GEO database, which helped us gain a better understanding of the mechanisms by which Tan IIA can perform the abovementioned functions. The results showed that the majority of the genes whose expression levels changed as a result of Tan IIA therapy were targets of the PI3K-AKT signaling pathway. This signal pathway can activate HIF-1α and HIF-2α, as well as stimulate the production of VEGF and other factors, hence boosting the growth of blood vessels (Ding et al., 2022). It is also a probable compensatory mechanism of drug resistance to Sorafenib in patients with liver cancer (Zhang et al., 2018). Additionally, it has also been shown that sustained endothelial activation of the PI3K-AKT signal pathway induces the formation of architecturally aberrant blood vessels that mimic the abnormalities of tumor vasculature (Kobialka et al., 2022). Therefore, Tan IIA enhances the normalization of tumor blood vessels and increases the susceptibility of hepatocellular carcinoma cells to Sorafenib through a credible candidate for the molecular mechanism.

**FIGURE 7:**
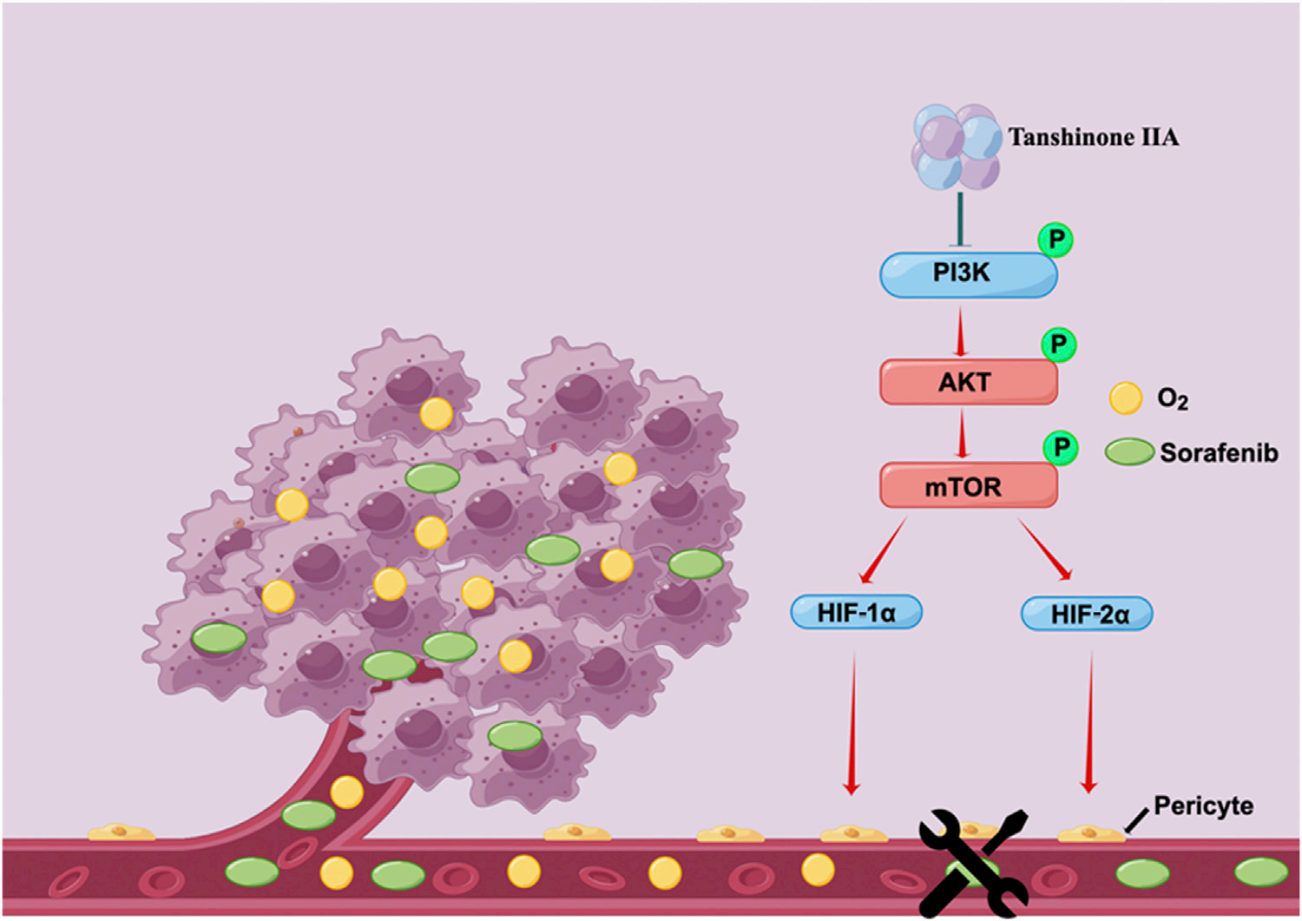
Schematic depiction of the underlying mechanisms through which Tan IIA restores the typical structure of tumor blood vessels by modulating the activity of the PI3K-AKT signal pathway improves the hypoxic microenvironment of liver cancer cells and hence increases their susceptibility to Sorafenib (drawn by Figdraw, www.figdraw.com).

Our research demonstrates that Tan IIA can restore the integrity and permeability of tumor blood vessels, thereby ameliorating the hypoxic milieu of hepatocellular carcinoma and increasing Sorafenib’s anti-cancer action. In addition, Tan IIA decreases the expression of HIF-1α and HIF-2α by inhibiting the PI3K-AKT signal pathway, which is a potential molecular explanation for the above-mentioned activities of Tan IIA (Figure 7).

## Data Availability

The datasets presented in this study can be found in online repositories. The names of the repository/repositories and accession number(s) can be found below: https://www.ncbi.nlm.nih.gov/geo/query/acc.cgi?acc&equals;GSE85871.
